# Lymphocytic Hypophysitis in a Patient With Suspected Syndrome of Inappropriate Antidiuretic Hormone Secretion (SIADH)

**DOI:** 10.7759/cureus.30178

**Published:** 2022-10-11

**Authors:** Jamie Thomas, Aakangsha Jain, Hernando Chong

**Affiliations:** 1 Osteopathic Medicine, Nova Southeastern University Dr. Kiran C. Patel College of Osteopathic Medicine, Fort Lauderdale, USA; 2 Family Medicine, Baptist Health South Florida, Miami, USA

**Keywords:** pituitary disorders, autoimmune disorder, hypothyroidism, hyponatremia, siadh, lymphocytic hypophysitis

## Abstract

Lymphocytic hypophysitis (LH) is a rare, autoimmune condition that presents with a range of symptoms that must garner the attention of medical practitioners. Clinically, it is characterized by symptoms of a compressive sellar mass with varying degrees of hypopituitarism due to chronic inflammatory infiltrate of the pituitary gland. It is often seen in women in their third trimester or postpartum and is associated with other autoimmune phenomena. Our case report describes a 73-year-old female with a past medical history of hypothyroidism and hypertension, who presented with continued intermittent dizziness, fatigue, and mild subjective hearing loss for the past several months. She was referred to the emergency department due to a sodium level of 119 and was initially diagnosed with syndrome of inappropriate antidiuretic hormone secretion (SIADH). The patient was treated accordingly; however, she failed to show signs of improvement. Due to her clinical presentation, imaging studies, and laboratory results, the patient was suspected to have LH, which was confirmed with the improvement of her symptoms after treatment with steroids. Because of the rare occurrence and possible atypical presentation of LH, this case illustrates the importance of maintaining a high index of clinical suspicion when diagnosing a patient with an unknown cause of hyponatremia, especially in patients with coexisting autoimmune disorders.

## Introduction

Lymphocytic hypophysitis (LH) is a rare, autoimmune condition in which the pituitary gland is infiltrated by lymphocytes. It often presents in women in their third trimester or within six months postpartum. Patients develop either lymphocytic adenohypophysitis or infundibulo-neurohypophysitis, with a small percentage developing both, resulting in lymphocytic panhypophysitis [1]. Features of hypopituitarism present due to inflammation of the pituitary gland through the infiltration of the gland by T and B lymphocytes [2]. As a result of the chronic infiltrate, this disease presents with two predominant types of symptoms: a varying presentation of endocrine symptoms including hyperprolactinemia, anterior pituitary hormone deficiencies, and central diabetes insipidus or symptoms resulting from mass effect [3]. The degree of lymphocytic infiltration corresponds to the degree of enlargement of the pituitary gland, which in turn places pressure on nearby structures, causing headaches, visual problems, and subclinical hypopituitarism [2]. 

Hypopituitarism has been shown to cause hyponatremia secondary to syndrome of inappropriate antidiuretic hormone secretion (SIADH). Mechanistically, due to a loss of adrenocorticotropic hormone (ACTH) secreting corticotrophs, there is secondary adrenocortical insufficiency and a loss of the regulatory effects of cortisol on arginine vasopressin [4]. Due to the rarity of this disease, diagnosis and treatment can be challenging. Depending on the presenting symptoms, treatment options include observation, glucocorticoids, or surgery [3]. Here we present a unique case of LH in a 73-year-old female with hypothyroidism who presented to the hospital with severe hyponatremia initially diagnosed as SIADH. This case demonstrates the importance of maintaining a high index of suspicion for this autoimmune condition as it can present atypically with a wide range of symptoms.

## Case presentation

Here we present the case of a 73-year-old female with a past medical history of hypothyroidism and hypertension who presented to her primary case physician (PCP) on March 15, 2021, with continued intermittent dizziness, fatigue, and mild subjective hearing loss for the past several months. On physical exam, the patient was also found to have gait instability. Due to her complaints and physical exam findings, her PCP ordered an MRI of the brain. 

**Figure 1:**
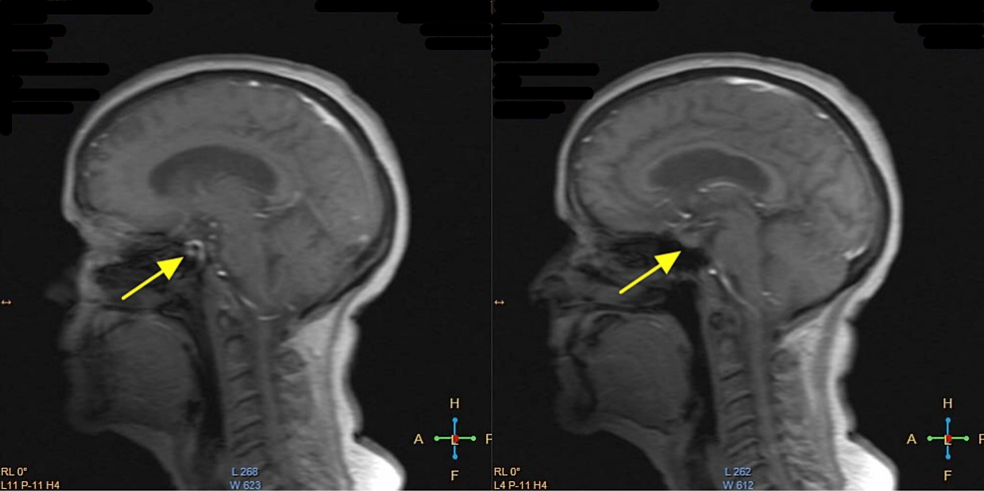
Patient's brain MRI showing pituitary gland enlargement.

On May 17, 2021, the patient followed up with her PCP to discuss the results of her MRI of the brain with and without contrast, which demonstrated diffuse brain atrophy with periventricular and juxtacortical changes compatible with small vessel ischemia. There was also an enlarged appearance of the pituitary gland (Figure 1). The patient also complained of continued somnolence, weight loss, generalized weakness, mental slowness, and dizziness and was referred to neurology. 

**Figure 2:**
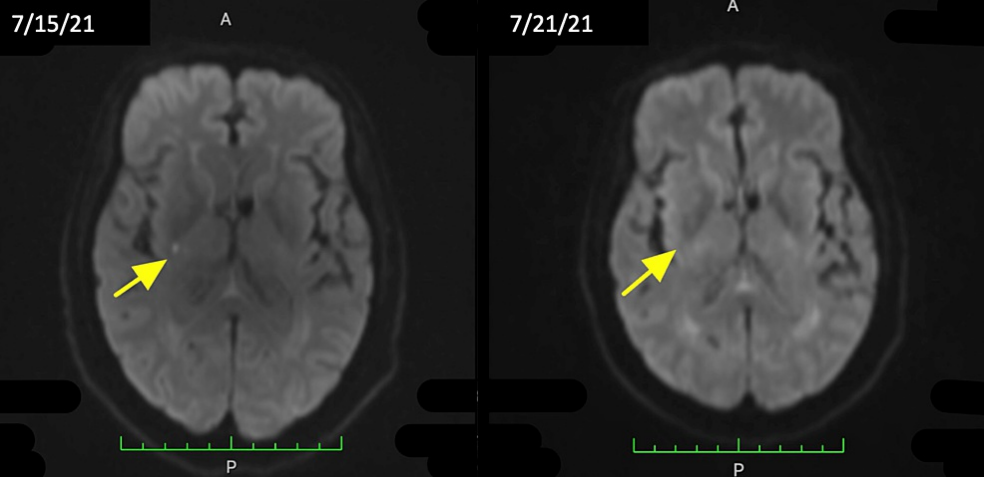
Patient's brain MRI showing punctate focus of restricted diffusion in the right posterior limb of the internal capsule consistent with an acute lacunar infarct (left). Infarct no longer seen (right).

On July 15, 2021, the patient’s neurologist ordered a repeat MRI of the brain due to enlarged pituitary on the previous exam. Per radiology, MRI of the brain with and without contrast showed punctate focus of restricted diffusion in the right posterior limb of the internal capsule consistent with an acute lacunar infarct (Figure 2), and an enlarged pituitary gland with no differential enhancement or stalk deviation. Radiology also advised to correlate clinically due to suspected possible diagnoses such as pituitary hyperplasia, symmetric pituitary adenoma, or LH. 

Once laboratory results returned on July 21, 2021, the patient’s neurologist referred her to the emergency department (ED) due to a sodium level of 119. At the time of presentation, the patient was oriented but had difficulty with word finding and answered questions inappropriately. Her labs in the ED revealed a sodium of 122 and potassium of 3.0. Nephrology was consulted and the patient was started on hypertonic saline at 20 cc/hour. She was admitted for further work-up and management of hyponatremia and altered mental status. A brain CT without contrast in the ED showed no abnormalities. However, per neurology, a stroke was unable to be ruled out given her recent acute transient ischemic attack (TIA) and word-finding difficulty. 

A stroke was eventually ruled out as the lacunar infarct seen on MRI on July 15, 2021, was no longer seen on repeat MRI on July 21, 2021 (Figure 2). However, the MRI redemonstrated a pituitary enlargement. The patient’s presumed diagnosis at this time was SIADH given her inpatient laboratory results demonstrating a urine sodium of 98 and urine osmolality of 293, as well as pituitary enlargement, recent administration of hydrochlorothiazide (HCTZ), and concurrent use of escitalopram. Throughout the hospital course, the patient was continued on fluid restriction and was discharged on July 23, 2021, once sodium stabilized at 131. HCTZ was discontinued and escitalopram was continued as per nephrology recommendation, along with the addition of sodium tablets. The patient was referred to follow-up with outpatient neurology and endocrinology.

On August 24, 2021, the patient was once again referred to the ED from her endocrinologist as she presented with vomiting and a sodium level of 121. The patient was also seen in the hospital and by her PCP twice in the past month for near-syncopal episodes and continued hyponatremia despite compliance with her salt tablets. Due to her recent admission for suspected SIADH, pituitary insufficiency and SIADH remained her top two differentials. The next day, the patient's endocrinologist assumed care with the initial impression that the primary problem was pituitary insufficiency with secondary adrenal insufficiency due to LH. He noted her laboratory results strongly confirmed this diagnosis as her cortisol level was 6, sodium was 121, gonadotropin was 0 despite menopause, thyroid stimulating hormone was 1 with very low free T4, and insulin-like growth factor level was low, all classically consistent with global pituitary hypofunction. In order to confirm her diagnosis, the patient was admitted and started on stress doses of hydrocortisone (Solu-Cortef 100 mg intravenous push injectable every 8 hours) along with a continuous saline infusion. The patient’s sodium responded within 24 hours and dramatically improved following the treatment. She was discharged with a therapeutic dosage of prednisone (10 mg three times a day) and continued levothyroxine (100 mcg once daily). 

At her follow-up appointment on September 27, 2021, the patient’s sodium was 137, which prompted a reduction of her prednisone from 5 to 2.5 mg once a day. However, she was unable to tolerate the reduced dosage as this led to reflux symptoms including polydipsia, polyphagia, and polyuria. She was returned to 5 mg of prednisone and her symptoms subsequently improved. 

## Discussion

LH is the most common form of primary hypophysitis when compared to other etiologies such as granulomatous, xanthomatous, and IgG4-related hypophysitis. LH has an incidence of one in 9 million and a striking predilection for women in their third trimester or first six months postpartum in 67% of cases [2]. The pituitary increases in size by about 30% during pregnancy due to estrogen-driven hypertrophy and hyperplasia of the lactotrophs, which may lead to the release of pituitary antigens. Pregnancy can also unmask a latent pituitary insufficiency and thus bring more cases to medical attention [5]. 

In prior systematic reviews conducted on patients with LH, it was found to have a positive association with other autoimmune conditions in about 20% of cases, including Hashimoto’s thyroiditis, autoimmune polyglandular syndrome type 2, Graves’ disease, and systemic lupus erythematosus [3,5]. Current research demonstrates the coexistence of thyroiditis and lymphocytic cell infiltration of the anterior pituitary may not be incidental, and is possibly due to an onset of autoimmune reaction to thyroid and pituitary antigens released during puerperal involution of the glands [6]. Our patient presented with a unique case due to being an elderly postmenopausal female with prior existing autoimmune thyroid disease. 

Diagnosis of LH poses a challenge due to the distinction between rare cases of LH and the more commonly presenting pituitary adenomas. At the time, such distinction can only be achieved with certainty through biopsy of pituitary tissue. Even when using MRI studies, a majority of cases are misdiagnosed as pituitary adenomas [5]. MRI findings for LH are extremely variable as imaging findings can show thickened pituitary stalk, diffuse pituitary expansion, homogeneous enhancement, loss of the neurohypophysis bright spot on T1 imaging, and abnormal enhancement of the infundibulum [7]. However, in LH, the typical pre-contrast MRI findings include a symmetric enlargement of the pituitary gland [5]. Our patient’s MRI showed vague signs of an enlarged pituitary gland with no differential enhancement or stalk deviation. As such, clinical features, endocrinological assessment, immunological markers, and imaging studies are often used to make a clinical diagnosis as a biopsy or surgical resection of suprasellar or sellar tissue carries a high risk and is not a practical approach [5,8]. 

The amount of lymphocytic infiltration in autoimmune hypophysitis relates directly to the proportion of enlargement seen in the pituitary gland. This enlargement adds pressure on nearby structures causing what are often the most common and initial complaints: headaches, dizziness, and visual disturbances [2]. The next most common symptoms include signs of partial or complete hypopituitarism, which are caused by the autoimmune destruction of the pituitary gland. Although LH commonly affects the anterior pituitary rather than the posterior pituitary and normally shows symptoms of anterior pituitary hormone deficiency, central diabetes insipidus has been frequently associated with LH [5]. This can be due to either the compression of the posterior lobe and infundibular stem or the direct extension of the lymphocytic infiltrate into the neurohypophysis [9]. Our patient was unique in that she was initially diagnosed with SIADH due to the finding of severe hyponatremia and elevated urinary sodium along with the use of hydrochlorothiazide and escitalopram. This can likely be explained by the hypopituitarism causing a decrease in ACTH secretion leading to secondary adrenal insufficiency. The ensuing loss of cortisol decreases its negative inhibitory effects on vasopressin, which increases the effect of antidiuretic hormone, leading to the hyponatremia that was seen in our patient [4]. 

In order to confirm our patient’s diagnosis and rule out SIADH as a significant contributing factor to her symptoms, the patient was admitted and started on stress doses of hydrocortisone along with saline infusion. Sodium should rapidly improve if pituitary insufficiency was suspected, but would fail to improve if SIADH was the cause of her symptoms. The patient’s sodium responded quickly to treatment, which officially confirmed the diagnosis of LH. In current practice, the treatment of LH is only symptomatic and includes reducing the size of the pituitary mass and/or repleting the pituitary hormone deficiencies. Pituitary mass reduction can be accomplished through lymphocytic drugs (glucocorticoids, azathioprine, or methotrexate), radiotherapy, or surgical resection of the pituitary gland. Current literature favors beginning with lymphocytic drugs as this is the least invasive approach [10]. Our patient was discharged on steroids, tapering the dose in four to six weeks, followed by a reassessment with the goal of inducing remission.

## Conclusions

Although coined to be the most common type of hypophysitis, LH is a rare autoimmune condition most commonly seen in women in their third trimester or postpartum that continuously proves to be a disorder that is often difficult to diagnose. This can be due to the possibility of revealing itself in a wide range of ages and its ability to present itself with various symptoms as it can affect the adenohypophysis, neurohypophysis, or both, along with symptoms due to an enlarging pituitary mass. In this report, we present a unique case of an elderly patient who presented with symptoms of severe hyponatremia that was diagnosed with underlying LH. Her atypical symptoms presented a challenge to her providers as they considered more common etiologies of hyponatremia. The patient’s symptoms were successfully controlled with steroids; however, her condition relapsed while attempting to taper her dosage. 
